# Public Address to the Members of the Illinois State Medical Society and the Citizens of Bloomington, on the Nature of Medical Science and Its Relations to the Community

**Published:** 1865-05

**Authors:** N. S. Davis

**Affiliations:** Chicago, Illinois


THE
### CHICAGO MEDICAL EXAMINER.

N. S. DAVIS, M.D., EDITOR.

VOL. VI. MAY, 1865. NO. 5.

### Original Contributions.

ARTICLE XVI.

### PUBLIC ADDRESS TO THE MEMBERS OF THE ILLINOIS STATE MEDICAL SOCIETY AND THE CITIZENS OF BLOOMINGTON, ON THE NATURE OF MEDICAL SCIENCE AND ITS RELATIONS TO THE COMMUNITY. DELIVERED MAY 2D, 1865.

BY N. S. DAVIS, M.D., Chicago, Illinois.

PROFESSIONAL BRETHREN AND FELLOW-CITIZENS:

In compliance with a special request from the McLean Co. Medical Society, I rise to address you on the present occasion. To select a subject for discussion which shall be interesting and profitable, both to the profession and the community, is not an easy task. The true nature of medical science, and its relations to the welfare of civilized communities, is perhaps less understood than that of any other important department of human study. Many seem to regard it as little more than a collection of theoretical dogmas, in regard to the nature of diseases and the mode by which medicinal agents can effect a cure. Hence they often speak of *systems* of medical practice, such as the Botanic, Eclectic, Hydropathic, Homœopathic, Allopathic, &c.; thereby clearly indicating their belief in the idea that all diseases are governed by some one law or hypothetical rule; and that all remedies are to act in accordance with some correspondingly general principle. Many more seem to regard all medical knowledge as a kind of intuitive mystery, capable of being acquired and understood by certain individuals, without any regard to their previous mental discipline or acquirements.

The first of these classes are easily captivated by every new theory, whether it be the simple imaginary law that "like cures like;" or the absurd reversal of all the laws of nature embodied in the *Hahnemannic* proposition that the potency of medicinal agents is increased in proportion to their dilution and attenuation. While the second are quite as likely to look with awe on the mountebank performances of the bold charlatan who claims to cure disease by a touch or a word; or to place implicit confidence in the pretensions of any ignorant man or woman who claims to cure diseases of every kind by some specific, professedly obtained from Indians, or other equally ignorant sources.

Comparatively few clearly appreciate the fact, that true medical science consists in such an extension and development of the various branches of the natural sciences, as will embrace a knowledge of the morbid or diseased conditions of living beings in addition to the healthy; and also a knowledge of the various agents capable of influencing such morbid conditions. Thus practical medicine; the art of curing or mitigating diseases; which constitutes the chief employment of the physician, rests directly upon pathology and materia medica. The first embodying a knowledge of the nature of diseases, and the second, of the particular means for their treatment.

But pathology is, itself, developed directly from an extension and combination of the facts derived from the well-known departments of natural science termed anatomy, physiology, organic and animal chemistry, and natural history; while the materia medica is equally the offspring of botany, chemistry, minerology, and natural philosophy. The art or practice of surgery is founded, if possible, still more directly upon the facts and principles of anatomy, physiology, and mechanics. Indeed, there is not a single department of natural science or philosophy that does not contribute to, or constitute a part of, medical science. Disease, instead of being a mysterious something—an intangible essence or spirit of evil, introduced into the human system through some channel, and capable of moving from one organ to another, is simply an alteration, either in the composition or action of some part of the solids or fluids of the body. Instead of being uniform in its character, capable of being treated by some specific remedy, or in accordance with some one theoretical law of cure, it is as variable in its symptoms, tendencies, and results, as are the atmospheric, hygienic, and mental influences that surround man in civilized life. And hence, a knowledge of the nature and influences of climate, topography, season of the year, diet, drinks, and mental habits, are as essental to the enlightened physician as a knowledge of anatomy or physiology. The science and art of medicine may be defined, therefore, to be the application of the facts and principles embodied in the various departments of natural, economical, and metaphysical sciences to the elucidation, prevention, and cure of diseases.

It is, in fact, the highest development of science applied to the noblest of temporal objects, namely, the prevention and alleviation of human suffering, and the prolonging of human life. Hence, in all ages of the world, the most eminent and successful cultivators of science have been educated members of the medical profession. If such is the nature, extent, and objects of medical science, it is hardly necessary to add, that its successful study and practical application require a mind naturally capacious, evenly developed, and thoroughly disciplined by systematic exercise.

Founded, as it is, on the various departments of the physical sciences, the principles and practice of medicine derives improvements and progress from every advancement made in those departments of human knowledge. Thus the advancements in analytical and organic chemistry; the greater perfection and more extended application of the microscope, and other philosophical means of investigation, have added immensely to the domains of medical science, during the last thirty years. By combining the aid of the knife of the anatomist; the test glass and crucible of the chemist; and the magnifying glasses of the microscopist, with the experiments of the physiologist; the relations, composition, properties, and atomic organization of every part of the human system has been fully brought to light. The same means of investigation are also directed to the examination of all the physical circumstances, whether atmospheric, terrestrial, dietetic, or social, that surround man as a social being, and which may exert an influence on his health and life. Hence the science and art of medicine has not only received great additions to its facts and appliances for the relief of human suffering, during the last half century, but it is still advancing *puri passu,* with the progress of all other departments of the natural and physical sciences. Those who cavil at our profession, often allude to the many changes in medical theories and opinions as evidence of the instability and worthlessness of our science. As well might they cavil at astronomy because the number of stars and the rules of mathematical calculations which it embraces to-day, do not correspond with those known to Copernicus in the sixteenth century; or at chemistry because the extensive works of a Lavoisieur, a Davy, a Liebig, a Henry, and a Draper, of the present century, could hardly be recognised as belonging to the same department of human learning as the vague doctrines of alchemy once taught in the renowned school of Arabia.

So long as man continues to inhabit the earth, with his living sensitive organization, exposed to the ever changing physical and mental influences that surround him in the various relations of life; and our knowledge of the nature, composition, and mode of action of these influences is capable of extension; so long will the science of medicine continue its onward progress. The truly scientific and rational physician is, in the highest sense, a student of nature. He takes the living human body, combining in its organization every principle of mechanics; every form of structure; and every manifestation of function to be found in the whole series of inferior animal organizations; and, as an anatomist, he separtes its bones, ligaments, muscles, nerves, bloodvessels, &c., noting carefully the exact relation each bears to the other; as a physiologist, he studies particularly the function or office that each part performs, and the influence it exerts on all the other parts; as a chemist, he analyzes the solids and fluids, ascertaining the ingredients of which each part is composed, and, of course, the materials necessary for its nutrition and growth; as a naturalist, he learns the laws of its development by watching the order of its growth, from the primary germ to the complex perfection of maturity, and compares each successive stage of development with the growth of corresponding parts in other species of animals, to render a knowledge of its functions more complete; as a geologist, meterclogist, social and political economist, he studies the effects of soil, climate, atmospheric changes, seasons, diet, drinks, social and municipal conditions, on each and all of its several structures and functions. He thus, not only learns the laws and conditions of its life and health, but also, that in all these combined, ever acting, and yet ever varying influences, the effects of some will be exaggerated while others are deficient, thereby destroying the equilibrium or healthy standard of action in some one or more of the organs of the body. And this will constitute disease. Studying this, he soon finds that, so far from being a uniform process or morbid condition, it may be one of excitement, depression, or perversion, according to the nature and intensity of the cause, compared with the temperament of the individual patient. Thus, by the same careful examination; the same modes of study; and the same inductive reasoning, as is pursued in the cultivation of any other science, the physician gains a reliable knowledge of the causes, symptoms, and nature of diseases. He follows no more theoretical dogma, but recognizes his patient as a living, delicate, sensitive organization, constantly surrounded and acted upon by a great variety of physical and mental influences, each capable of producing its own morbid impression. Having fully appreciated, not only the existence and phenomena of diseases, but also the almost endless variety of forms and tendencies they exhibit, he next ransacks the whole fields of chemistry, botany, mineralogy, and natural history for materials capable of allaying vital action when excited; of stimulating it when enfeebled; of correcting it when perverted, that he may become the intelligent and efficient helper and guide of nature in regaining the natural standard of action, or what we call health.

Here may be clearly seen one of the broad distinctions between true medical science and the various *pathys* or so-called special systems of medicine. The first is emphatically a study of nature, mutually receiving and imparting facts with every other department of science, and, consequently, ever advancing; while the second are simply so many arbitrary human dicta, incapable of either expansion or improvement. It matters not whether they assume the form of the obsolete Thompsonian axiom, that "heat is life, and cold is death;" or the Botanic maxim, that "vegetables growing upward tend to life, and minerals downward tend to death;" or the present more popular, but equally fallacious, expression of the Homœopath, "*similia similibus curantur;*" they are alike Procrustean beds, to which their disciples are ever vainly striving to make all the facts in nature and philosophy fit. My leading object in thus calling your attention to the true nature of medical science, is to make more apparent the relations it bears to the various important interests of civilized society. Many, I might say the vast majority of mankind, regard medical science and art as having no other object than the direct treatment of the sick. But this, though an object of great and almost sacred importance, is not the only one that should challenge the attention and enlist the cordial coöperation of every enlightened community. To cure disease, and thereby alleviate human suffering and prolong human life, is, indeed, a beneficent—a noble work. But to *prevent* both disease and suffering is more beneficent, as well as far more desirable. I do not exaggerate in saying that the latter is as legitimate a part of the objects of those engaged in the study and practice of medicine as the former. From the very nature of their studies they become familiar with the causes of disease, the modes by which they are developed, the laws governing their diffusion, and the means for preventing or neutralizing them; and, from an early period in the history of medicine, the knowledge thus acquired has been aggregated under the head of hygiene or sanitary science, and made more or less subservient to the welfare of man. How many are there, in this or any other community, who realize the actual extent to which they are enjoying benefits derived from this department of medical knowledge? To whom is the civilized world indebted for the knowledge which has led to the widening, cleaning, and sewering of the streets of all the densely populated cities; by which the ratio of mortality has been reduced twenty-five, and, in some instances, fifty per cent? To whom is it indebted for improvements in the construction, warming, and ventilation of public and private buildings, workhouses for the poor, hospitals for the sick, asylums for the insane, and prisons for the criminal? To whom is it indebted for a knowledge of the laws governing the development and spread of contagions and infections, on which are founded all enlightened quarantine and police regulations for protection against the spread of severe and fatal diseases? To whom is it indebted for that knowledge which has led to such improvements in the preparation, preservation, and transportation of food as has banished that former scourge of the ocean, *scurvy*, from every well-regulated ship; and greatly facilitated the movements, efficiency, and comfort of armies, whether on the land or the sea? To whom is it indebted for the means of securing almost every man, woman, and child against an attack of that most dreaded of all diseases, the small-pox? For all these, and much more, the world is indebted, almost exclusively, to the cultivators of medical science; to that profession whose true votaries may be seen at midnight, as well as at noonday, not only climbing the hills, traversing the lonely prairies, and fording the streams, to administer relief to the sick, whether in the mansion of the rich or the hovel of the poor, but also in the laboratory of the chemist; the alleys, cellars, and garrets teeming with filth and infection, the very chambers of pestilence; and even the charnel-house of the dead, quietly seeking out and giving to the world that knowledge which will aid in preventing as well as curing disease.

If any of my hearers are skeptical in regard to the benefits that have been conferred on the human race, in the prevention of disease, by that department of medical knowledge termed hygiene and sanitary science, let them compare the London of to-day with the London of the sixteenth century. Let them compare the ravages of the plague and small-pox in the sixteenth, with the effects of the same, or any other diseases, in the nineteenth century. Let them compare the mortality and suffering from scorbutic diseases on the sea, during the former with the latter period of time. Let them compare the modern asylum, or hospital for the insane, with the dungeons and prisons in which this unfortunate portion of our race were literally chained, until death relieved them of their suffering. It would set at nought the skill of the mathematician to estimate either the number of lives saved, or the amount of human suffering prevented, by the influences to which we have briefly glanced, during the last half century alone. And, yet, the knowledge actually furnished by the profession has been so imperfectly applied, both by individuals and by municipal authorities, that the beneficial results are scarcely half developed. Neither the sanitary police, nor the systems of sewerage, in the cities and densely populated districts, are what science and the happiness of the people require. In the construction of dwellings, and the physical training of children, the most important and well-known laws of health are violated daily and almost universally. It has been demonstrated that a certain number of cubic feet of atmospheric air is necessary for the maintenance of a healthy state of a single individual for a given number of hours. Yet, in the construction of dwellings, and especially of lodging-rooms, this fact is often overlooked. Not only in our cities, but all over these broad prairies, you may find in the low, garret-like chambers, bedrooms partitioned off so small, and so little ventilated, as to be incapable of supplying one-half of the air required by those sleeping in them, in order to promote full, vigorous health. Is it surprising that the inmates rise in the morning with feelings of languor, debility, mental dulness, and loss of appetite, instead of freshness and vigor? With their nervous functions depressed, and the blood impure from insufficient air, is it strange that they should frequently fall victims to typhoid and lung fevers in winter, or enteric fever and dysentery in summer? It is one of the most important and familiar facts in physiology, that the tissues of the human system can be increased in size, strength, and health, by judicious exercise, and impaired by the neglect of it; thus furnishing every parent and teacher with a reliable rule by which he may so guide the mental and physical training of children as to develope and strengthen the faculties that are small and weak, and retard the growth of those already in excess, and thereby establish that just equilibrium of development at maturity which is most conducive to health, long life, usefulness, and happiness. Yet, how often do we see the rule either entirely neglected, or its practical application reversed, both in the family and in the school; thereby keeping the community full of ill-balanced minds and unequally developed bodies, with an inexpressible amount of moral, intellectual, and physical suffering as the result. Again, medical science has long since furnished a clear distinction between food, or such substances, whether solid, fluid, or gaseous, as are capable of nourishing the various structures of the human body; and medicines or poisons which, though capable of producing certain effects on the functions of the system, are wholly incapable of nourishing, or becoming a healthy part of its structures. It has demonstrated the first to be essential as a daily supply to every individual in health; while the latter, though of great value in skilful hands, as agents for correcting morbid or diseased conditions, are necessarily disturbing and injurious if taken in a condition already normal and healthy. And yet large numbers in almost every community continue to use certain articles of this class habitually, and without any regard to sickness or health. I here refer more directly to the use of alcoholic drinks and tobacco. I am aware that large portions of the community, and even some members of the medical profession, regard these powerful agents as food, at least in a qualified sense; or, as one recent writer has styled them, "*accessory* food." But their position is neither sustained by facts nor sound reasoning. By the enlightened, it is not claimed that either alcohol or tobacco can furnish the slightest material for actual assimilation and natural addition to the tissues; but it is claimed that their presence in the system retards those changes by which the organic atoms, already constituting a part of the tissues, are disintegrated and removed. In other words, they retard the waste of the different structures, and thereby render the supply of ordinary food less necessary—thus, indirectly, supplying the place of actual food. This reasoning is plausible, and well calculated to deceive those who have only a superficial knowledge of the phenomena of life and organization. If the atoms composing the delicate structures of the physical man had been designed by the great Architect of nature to remain stationary or permanent, from day to day, then an atom *retained* would, indeed, be equivalent to an atom *supplied.* But, instead of this, it is found that constant change is not only the normal condition of every cell and fibre of our physical structures, but that every movement or phenomenon of life is the result of change. The evolution of caloric to supply warmth; the elimination of secretions, whereby the blood is kept pure; the digestion and assimilation of food; the circulation of fluids, and the contraction of muscles, are dependent on, and accompanied by, atomic changes in the tissues. Indeed, atomic change may be regarded as the law of life, as inexorably as stassis is that of death. When the atomic changes in the tissues take place with a certain degree of rapidity, all the resulting and accompanying phenomena and functions of life are natural, and health exists. If, in this condition, any agent, whether whiskey, tobacco, or anything else, capable of retarding those changes is introduced into the system, nothing is more logically certain than that the healthy equilibrium of action will be interfered with, and an unnatural or morbid state of the system induced. Hence the idea that retarding the atomic or cellular changes in the tissues is equivalent to a supply of new atoms or cells from the assimilative processes, is simply a most mischievous fallacy. There is, however, another broad distinction between real food and mere exhilarants and narcotics. The first always satiates or imparts the feeling of enough, while each dose of the latter only sharpens the appetite for more. Thus, the man who has eaten bread daily, is as easily and perfectly satisfied with a given quantity of it at the age of sixty as he was when weaned from his mother's breast; and so of every other article of food, whether solid, liquid, or gaseous. But who, after habitually using alcoholic drinks, or tobacco, even for a few months, was ever satiated with either? But it is no part of my present purpose to enter upon a discussion of the evil effects of either alcohol or tobacco. They are everywhere so apparent that, literally, "he who runs may read them." I simply wished to vindicate medical science from the responsibility of even an indirect endorsement of their use.

In the foregoing rapid and imperfect review of the nature, objects, and relations of medical science, it is made apparent to all, that such science, instead of being restricted in its objects to the alleviation and cure of disease, is most intimately interwoven with the social, the educational, economical, and sanitary interests of the whole civilized world. And here again comes a broad distinction between true medical science and the various special systems to which we have once before alluded. The latter are not only "Procrustean beds," to which their supporters are trying to made all the phenomena of disease fit; but they are fixed dogmas totally incapable of conducting their disciples one iota beyond the narrow sphere of treating disease itself in a specific direction. Suppose we take the so-called, fundamental principle of homœopathy, that "like cures like," and apply it to the work of sanitary improvements. Would it suggest the removal of filth, foul air, and infection, by sewers, ventilation, and antidotes, or would it require of its disciples an attempt to correct one species of infection by establishing alongside of it a small quantity of another as near like it as possible? Indeed, who ever heard of a single attempt made to improve the physical and sanitary interests of society, by the followers of any one of the empirical systems of medicine, from the days of Paracelsus to that of Hahnemann? If, however, true medical science is but a more extended development of all the physical sciences, and the application of the facts and principles they furnish, to the great work of preventing and alleviating human suffering, thereby bringing it into the most intimate and important relations to the welfare of the whole community, it necessarily follows that certain obligations concerning it, devolve upon the community at large. For instance, it is one of their highest duties to make liberal provision for the thorough mental development and education of all who wish to devote themselves to the arduous work of *our* profession. It is more especially and imperatively their duty, and that of their representatives in the Municipal and State Legislatures, to afford the profession every possible facility for increasing its knowledge of the nature, causes, and tendencies of disease. They should do this by legalizing the study of anatomy by dissections, under proper regulations; by providing for the more frequent and thorough performance of *post mortem* examinations; by providing for the thorough observation and record of all atmospheric and climatic changes in connection with the prevalence of epidemic and endemic diseases; and by the enactment and rigid enforcement of efficient laws for the registry of all births, marriages, and deaths. By such measures the profession would be enabled, within a very few years, to add greatly to the present stock of knowledge concerning the causes of all diseases, whether ordinary or extraordinary; and also, concerning the laws governing the development, diffusion, and destruction of such causes. This would immediately bring in return corresponding improvements in the sanitary welfare and happiness of all classes of people. And, in conclusion, we ask this enlightened audience, here, in the central part of this great central State of our glorious and inseparable Union, to interest yourselves, individually and collectively, in favor of carrying these measures into practical operation. Some of them, as the legalizing of dissections, and the registry of births, marriages, and deaths, have already been adopted by New York, and several of the New England States. But the remaining ones, which are of equal, and perhaps paramount importance, are still awaiting initial action. As the people of our own State have emphatically led the way in subduing the recent gigantic and wicked rebellion, let them also, in future, equally take the advance in those enlightened and peaceful measures, that are so directly calculated to promote the dearest and most important interests of mankind.

It is not for the benefit of the profession that I represent, that these and kindred measures are asked. Their adoption would only impose on us additional labors,—but it is for the benefit of yourselves and of your posterity. On this evening, when the whole nation is draped in mourning, for the tragic and cruel death of its late Chief Magistrate, and his earthly remains are on their way to their last resting place, I cannot take my seat without expressing my full sympathy with the national grief. Whatever differences of opinion may have existed in regard to particular measures of governmental policy, the late President was regarded, by all considerate men, as a sincere patriot and lover of his country, and the time and manner of his death will be sincerely and lastingly lamented by every virtuous mind. But let us find consolation in the hope that unholy ambition, unbridled passion, and reckless treason have reached the climax of their wickedness and crimes, and that returning peace, if it cannot restore to life our precious dead, will at least restore to our country an indissoluble union of hearts as well as States, with the same bright old star spangled banner of freedom to forever float over us.

